# Neurotoxin-Derived Optical Probes for Elucidating Molecular and Developmental Biology of Neurons and Synaptic Connections: Toxin-Derived Optical Probes for Neuroimaging

**DOI:** 10.1007/s11307-024-01954-6

**Published:** 2024-09-30

**Authors:** Rohini Bijjam, Susan Shorter, Alison M. Bratt, Valerie B. O’Leary, Vasilis Ntziachristos, Saak Victor Ovsepian

**Affiliations:** 1 Faculty of Engineering and Science, University of Greenwich London, Chatham Maritime, Kent, ME4 4TB UK; 2 Department of Medical Genetics, Third Faculty of Medicine, Charles University, Ruská 87, 10000 Prague, Czech Republic; 3 Chair of Biological Imaging at the Central Institute for Translational Cancer Research (TranslaTUM), School of Medicine, Technical University of Munich, 81675 Munich, Germany; 4 Institute of Biological and Medical Imaging and Healthcare, Helmholtz Zentrum München (GmbH), 85764 Neuherberg, Germany; 5 Munich Institute of Robotics and Machine Intelligence (MIRMI), Technical University of Munich, 80992 Munich, Germany; 6 DZHK (German Centre for Cardiovascular Research), Partner Site Munich Heart Alliance, Munich, Germany; 7 Faculty of Medicine, Ivane Javakhishvili Tbilisi State University, 0159 Tbilisi, Georgia

**Keywords:** Fluorescent probes, Fusion proteins, Optical imaging, Molecular trafficking, SNARE proteins, Retrograde transport, Advanced biomaterials

## Abstract

Botulinum neurotoxins (BoNTs) and tetanus toxin (TeTX) are the deadliest biological substances that cause botulism and tetanus, respectively. Their astonishing potency and capacity to enter neurons and interfere with neurotransmitter release at presynaptic terminals have attracted much interest in experimental neurobiology and clinical research. Fused with reporter proteins or labelled with fluorophores, BoNTs and TeTX and their non-toxic fragments also offer remarkable opportunities to visualize cellular processes and functions in neurons and synaptic connections. This study presents the state-of-the-art optical probes derived from BoNTs and TeTX and discusses their applications in molecular and synaptic biology and neurodevelopmental research. It reviews the principles of the design and production of probes, revisits their applications with advantages and limitations and considers prospects for future improvements. The versatile characteristics of discussed probes and reporters make them an integral part of the expanding toolkit for molecular neuroimaging, promoting the discovery process in neurobiology and translational neurosciences.

## Introduction

Optical imaging is among the oldest and most rapidly advancing research techniques [1, 2]. Bolstered by the expanding toolkit of fluorescent probes and reporters, optical imaging has transformed the outlook of biological interrogation, exposing fundamental life processes across a wide range of spatiotemporal scales [3–8]. Combined with improving methods for targeting various cells and subcellular compartments, optical probes and reporters have become an essential part of the revolution in biological imaging, facilitating groundbreaking discoveries, offering better biocompatibility with lower off-site effects and toxicity [9–13].

The tremendous potency and specificity of many biological toxins, due to their targeting and interference with selected molecules and functional mechanisms in different cell types, are particularly interesting for research of cellular and sub-cellular processes. Throughout their evolutionary journey, the adaptive pressure has diversified and refined biological toxins for selectivity, potency, and stability [14–17]. Botulinum neurotoxins (BoNTs, A-G serotypes) and tetanus toxin (TeTX) are the most lethal biological substances, causing botulism and tetanus, respectively [18–20]. These are large modular proteins produced by *Clostridium botulinum* and *Clostridium tetani*, comprised of the C-terminal binding and N-terminal translocation domains of the heavy chain (HC and HN, respectively) and proteolytically active light chain (LC) [18, 19, 21, 22]. The HC is responsible for the neurotropism of toxins, binding presynaptic terminals via dual, low- and high-affinity interaction with gangliosides and protein receptors on the surface membrane [21, 23, 24]. This step is followed by the internalization of toxins with translocation of their LC protease into the cytoplasm and cleavage of soluble N-ethylmaleimide-sensitive factor attachment protein receptors (SNAREs) in presynaptic terminals, resulting in the block of neurotransmitter release [18, 21, 25–27]. The subtle structural differences between various types of toxins determine the action site and intracellular faith, with paralytic effects of BoNTs primarily confined to their entry location in presynaptic terminals of the neuromuscular junction (NMJ) [28–31]. In contrast, TeTX, after the entry into nerve terminals of motor neurons (MNs), hijacks the retrograde transport machinery of axons to reach the central nervous system (CNS), shutting down inhibitory synaptic inputs onto motor neurons (MNs) and resulting in spastic paralysis [18, 21, 26, 32, 33].

**Fig. 1:**
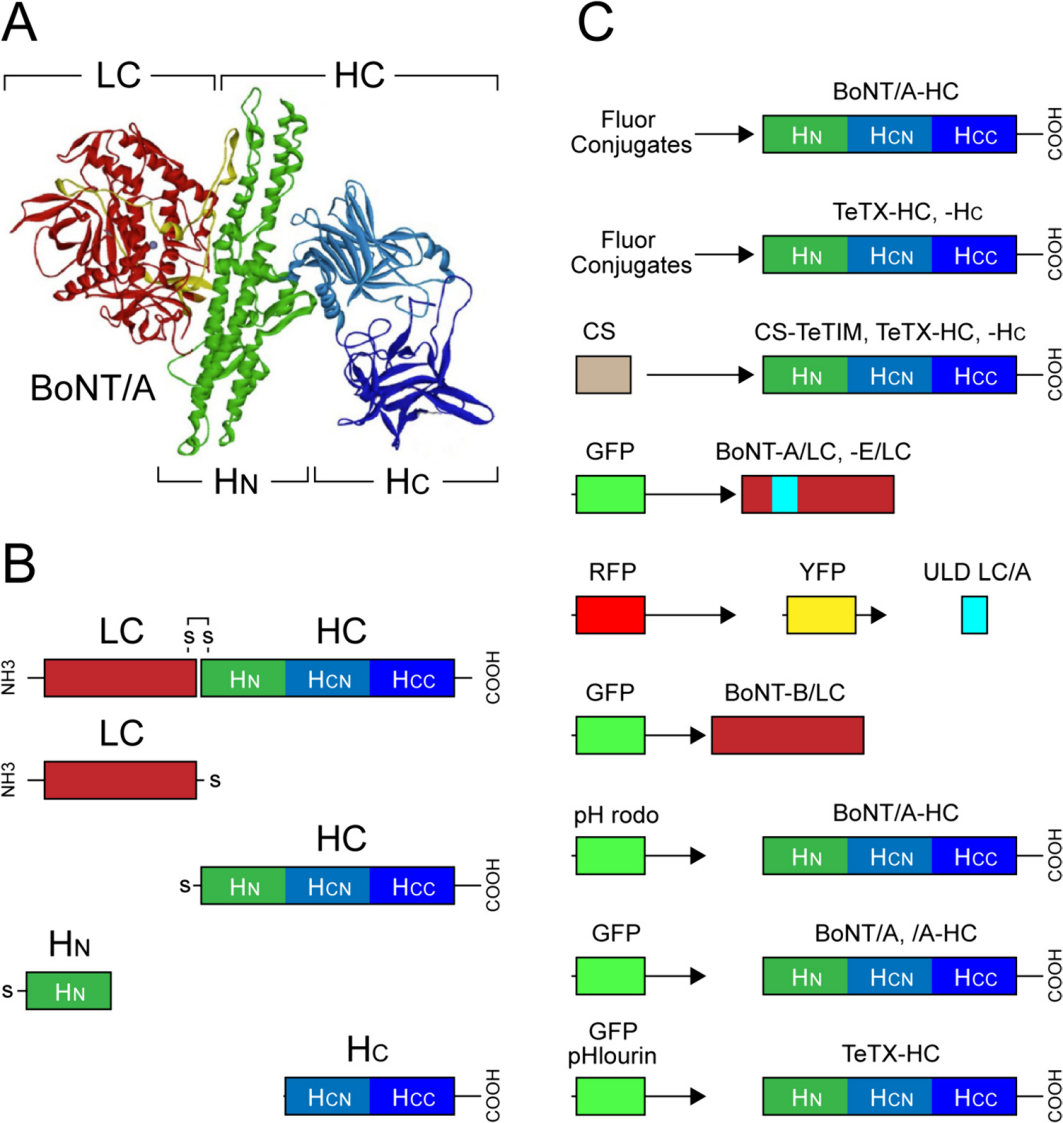
Structure and functional domains of neurotoxin-derived optical probes used for visualizing molecular, functional and developmental processes in neurons. (**A**) Crystal structure of botulinum neurotoxin type A (BoNT/A) with a colour-coded illustration of various sub-domains. The C-terminal binding domain (H_C_) of the heavy chain (HC) adopts a trefoil fold to bind the membrane surface of neurons, while the N-terminal jelly-roll motif of H_N_ facilitates the translocation of the light chain (LC) protease from the vesicular lumen to the cytoplasm of neuron. The LC contains the conserved HExxH motif characteristic of zinc-dependent proteases, which targets and cleaves SNAP-25 SNARE protein. Adapted with permission from [26]. (**B**) Schematic of BoNT/A di-chain (linked via S–S bonds), which is conserved across all BoNT (A-G) serotypes and TeTX. Break down of S–S bond in acidic environment of synaptic vesicles lead to release of LC into the cytoplasm of neurons. (**C**) Graphical representations of optical probes derived from BoNTs and TeTX or their sub-domains conjugated with fluorophores, or fused with reporter proteins (e.g. GFP, GFP-phlourin, YFP and RFP). Further details of structure and function of probes with various subdomains and their applications are described throughout this study

The tremendous potency and selectivity of BoNTs and TeTX for inhibiting transmitter release tart-taking in autonomic, sensory and motor functions have attracted much interest in their research and therapeutic use [20, 26, 34–38]. Targeting molecular cargo and genes to specific synaptic connections and neurons have also been of significant translational interest, with the increasing use of recombinant BoNTs and TeTX and fragments to deliver flours and reporter proteins for molecular and cellular neuroimaging. In this article, we present the state-of-the-art optical probes derived from BoNTs and TeTX and their fragments (Fig. 1). We revisit their applications in discoveries of the mechanisms of transmitter release at peripheral and central synapses, protein trafficking and metabolism, as well as results of mapping the hotspots of synaptic activity, formation and plasticity of neural networks and connections. We conclude by discussing the advantages and limitations of using neurotoxin-derived optical probes and consider areas and research directions for improving their future neuroimaging applications.

## Methods and Data Presentation

The authors searched and analysed the literature using scientific databases such as PubMed and ScienceDirect. Where appropriate, Google Scholar, Academia and ResearchGate have been utilized as additional sources of information. Keywords used for the search were 'fluor-labelled botulinum toxin', 'fluor-labelled tetanus toxin', 'BoNT fluorescence probes', 'TeTX fluorescence probes', 'molecular imaging with labelled BoNTs', molecular imaging with labelled TeTX', 'BoNT fusion protein for imaging', 'TeTX fusion protein for imaging' and 'visualizing synaptic functions with fluor-labelled toxins'. The reference list of articles was scanned to identify information relevant to the current analysis. A summary of all references was drafted, followed by thematic grouping and manuscript writing. Figures are prepared using Adobe Illustrator Artwork 16.0 of the Adobe Creative Suit version 6.0. The table has been generated using Microsoft Word. EndNote X8.2 was used for citations, with references formatted per Journal Guidelines.

## Mapping Subcellular Distribution and Action Sites of Neurotoxins

**Fig. 2:**
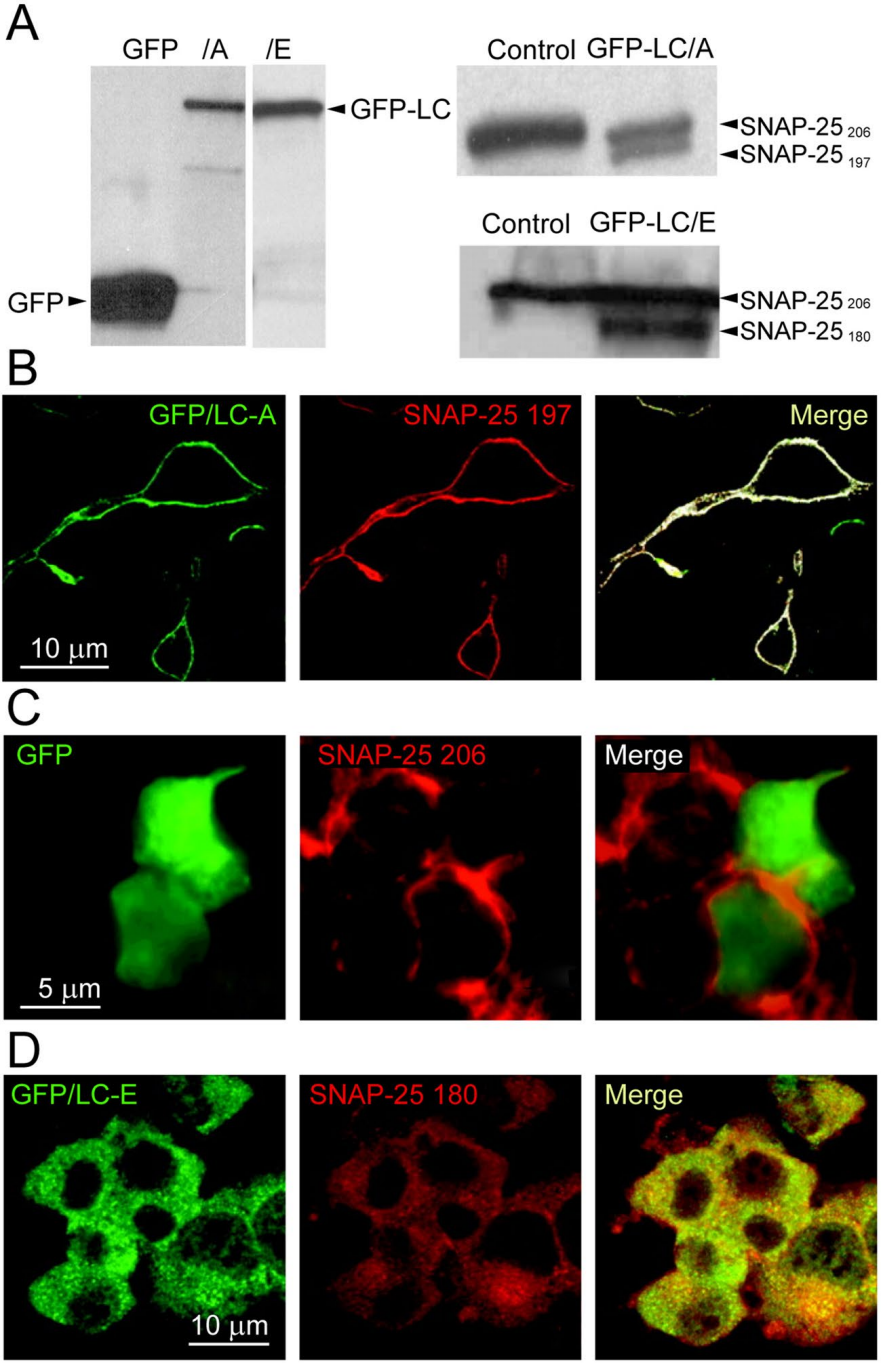
Visualizing subcellular distribution and action sites of BoNT -LC. (**A**) GFP-LC/A and GFP-LC/E expression (left) and validation of proteolytic activity by cleaving SNAP25_206_ into SNAP25_197_ and SNAP25_180_, respectively (right). (**B**) SNAP25_197_ and BoNT/A-LC colocalize in the plasma membrane. Differentiated cells expressing GFP-LC/A were stained with antibodies to GFP (green) and SNAP25_197_ (red). White colour on merged imaging indicates areas of colocalization of the GFP-LC/A with substrate SNAP25. (**C**) Non-targeted GFP expressing PC12 cells stained with antibodies to GFP (green) and SNAP25_206_ (red). Note the absence of colocalization of the two proteins. (**D**) SNAP25_180_ and BoNT/E-LC are cytoplasmic proteins. GFP-LC/E showed cytoplasmic localization with nuclear exclusion. Cells displayed rounded morphology and absence of neurites even in differentiation media. PC12 cells were stained with antibodies to GFP (green) and SNAP25_180_ (red). Adapted with permission from [42]

In mouse spinal cord cell culture, BoNT/A cleaved SNAP-25 was present > 80 days post-treatment, while SNAP-25 fragments produced by BoNT/E could be detected up to 1 week [39, 40] (Table 1). These differences correlate with the subcellular localization of BoNT/A and /E LCs, as revealed by the fusion protein of GFP with LCs (GFP-LC). Rat PC12 studies showed that GFP-LC/A labels the plasma membrane while GFP-LC/E is located mainly in the cytoplasm [41, 42] (Fig. 2A-D). Using confocal imaging and Western blotting, authors verify clustering of GFP-LC/A with cleaved SNAP25-197 on the surface membrane [42]. Site-directed mutagenesis has identified a unique AA sequence within the N-terminus of BoNT/A-LC targeting it to the plasma membrane, while the dileucine motif in the C-terminus guided the LC to the degradation route in lysosomes [42].

**Table 1:** Summary table of principal neurotoxin-derived optical probes discussed in this report, their biological origin and composition, fluorescence properties, and validation in models with research applications and references

Optical Probes	Originator Toxin / Fragments	Fluor-reporter	Model	Research Applications	Ref
BoNT/A LC-GFP; BoNT/E LC-GFP	BoNT/A, BoNT/E	GFP (a395-e475nm)	Rat PC12 cells	Action longevity of BoNT/A	[41]
BoNT/A LC-GFP; BoNT/E LC-GFP	BoNT/A, BoNT/E	GFP (a395-e475nm)	Mouse spinal cord neurons	Location of SNAP-25 cleavage, sorting for degradation	[42]
BoNT/A LC-GFP BoNT/E LC-GFP	BoNT/A BoNT/E	GFP (a395-e475nm)	Neuro-2A cells	Mapping intracellular localization of SNAP-25	[43]
BoNT/A1/A3 LC-GFP BoNT/A3/A1 HC-GFP	BoNT/A1 + A3 BoNT/A3 + A1	GFP (a395-e475nm)	Cultured primary Neuro-2A cells	Action duration and toxicity	[46]
Ubiquitin ligase domains of LC/A-RFP and LC/E-YFP	BoNT /A BoNT /E	Luciferase (a460-e640 nm)	N18 Neuroblastoma cells	Action longevity of BoNT/E	[51]
BoNT/A-LC-GFP, L428A/L429A	BoNT/A	GFP (a395-e475nm)	Human neuroblastoma cells; Rat PC12 cells; SH-SY5Y	Action longevity of BoNT/A, site of cleavage	[53]
BoNT/A-H_C_-Atto647N	BoNT/A	Atto647N (a625-e660 nm)	Rat hippocampal cell cultures	Mapping intracellular localization	[54]
BoNT/A-H_C_-Cy3; BoNT/A-H_C_-Oregon Green 488	BoNT/A	Cy3 (a554-e556 nm) Oregon Green 488 (a496-e524 nm)	Primary mouse cortical cells	Trafficking and delivery	[55]
BoNT/HC AlexaFluor-488; pH-rodo-BoNT/A-HC	BoNT/A	pHrodo Red (a560-e585 nm)	Mouse cultured hippocampal neurones	Targeting and delivery	[56]
BoNTA-Hc 544–1295-GFP, BoNT/A-Alexa-647	BoNT/A	GFP (a395-e475nm)	PC12 & NG 108–15 neurones Mouse NMJ and TS toxicity assay	Antitoxin therapy	[57]
BoNT/A-Hc-eGFP; BoNT/A-Hc-AlexaFluor-594; BoNT/A-Hc-AlexaFluor-680	BoNT/A	eGFP (a395-e475nm); AlexaFluor-594 (a590-e614 nm); AlexaFluor-680 (a679-e702 nm)	Mouse NMJ; rat spinal cord MN; Rat cerebellar granule neurones	Mapping intracellular localization, action site	[58]
BoNT/A-AlexaFluor-488 BoNT/E -Texas Red	BoNT/A BoNT/E	AlexaFluor-488 (a490-e525 nm); Texas Red (a561-e594 nm)	Rat hippocampal synaptosomes	Mapping intracellular localization of LC	[63]
TeTX Hc- FLASH; AlexaFluor-488, 546, 594,	TeTX	AlexaFluor-488 (a490-e525 nm)	Rat spinal cord motor neurones	Mapping intracellular localization	[68]
TeTX-Hc-GFP (PHluorin)	TeTX	GFP (a395-e475nm) [PHluorin]	Rat spinal cord motor neurones	Mapping intracellular localization	[67]
TeTX-Hc-GFP	TeTX	GFP (a395-e475nm)	Mouse levator auris longus (LAL) muscle	Mapping intracellular localization	[69]
TeTX-HC-em GFP	TeTX, HC (1066, 1093, 1109)	EmGFP (a395-e475nm)	Mouse NM explant; human striated muscle explants; NMJ of ALS (SOD1G93A) mice	Characterization of NMJ functions	[70]
BoNT/B-LC-GFP	BoNT/B	GFP (a395-e475nm)	Zebra fish – conditioning to specific stimulus	Physiological characterisation of brain pathways	[71]
TeTX Hc-GFP	TeTX	GFP (a395-e475nm)	Rat spinal cord neurons and organotypic cerebellar slices	Mapping intracellular localization; cargo delivery	[72]
TeTX-Hc-GFP	TeTX	GFP (a395-e475nm)	Orexin / EGPF transgenic mouse model	Mapping intracellular localization; tracking brain connections	[73]
TeTX-Hc-GFP	TeTX	GFP (a395-e475nm)	Brain slices of transgenic mice	Mapping intracellular localization	[74]
TeTX-LC-CFP	TeTX	CFP (a450-e550nm)	Rat embryonic cultured hippocampal neurones	Neurodevelopment, synaptic connections	[75]

Similar to PC12 cells, in Neuro-2A cells expressing GFP-LC/A, the protease was anchored to the substrate SNAP-25 at the plasma membrane, as evident from GFP-LC/A colocalizing with membrane-associated YFP-SNAP-25 [43]. In cells expressing four AA mutated SNAP25 (C85A, C88A, C90A, C92A) preventing palmitoylation at these critical cysteines anchoring SNAP-25 to the plasma membrane, ~ 80% GFP-LC/A was cytoplasmic. These data support the role of SNAP-25 in attaching LC/A to the plasma membrane, which is also the primary site of the cleavage of this SNARE protein by this protease [42, 44]. Accordingly, cleavage of SNAP-25 by LC/E between AA 180 and 181 with the release of SNAP-25 (1–180) into the cytosol altered the localization of GFP-LC/A from the plasma membrane to the cytoplasm. The re-distribution of LC/A to the cytoplasm was due to the cleavage of SNAP-25 because, in cells expressing catalytically inactive LC/E (R347A, R349F), GFP-LC/A remained anchored to the plasma membrane [43].

Differential localization of BoNT/LC has also been implicated in the heterogenious distribution and potency of BoNT/A variants, with BoNT/A2—A8 showing distinct efficacy, action duration and toxicity relative to BoNT/A1 [45]. BoNT/A3 has a shorter action duration and elicits distinct symptoms in mice at high doses compared to BoNT/A1 [20, 46]. Comparison of LC and HC of BoNT/A3, recombinant hybrids consisting of reciprocal LC and HC (BoNTA1/A3 and BoNTA3/A1) shows that both, the LC and HC of these chimeric molecules determine their toxicity in mice and in cultured neuronal cells. In contrast, the LC alone determines the action duration (see below). Protein alignment identified a previously unrecognized region within the LC/A3 relative to the other LC/A subtypes, which correlated with the intracellular LC localization, as revealed by the expression of enhanced GFP (eGFP) fusion protein with detoxified LC/A1 and LC/A3 in Neuro-2A cells. Unlike eGFP-LC/A1 localized on the surface membrane, eGFP-LC/A3 was mostly cytoplasmic [46]. Overall, these studies with optical probes derived from LC of various BoNTs suggest that the differential intracellular distribution of BoNT proteases determine their stability and faith, contributing towards potency and longevity of paralytic effects.

## Cellular and Molecular Determinants of the Lifetime of Protease

The half-lives of most proteins range from several hours to 4–7 days [47, 48]. In Digit Abduction Scoring (DAS) of rats, the paralytic effects of BoNT/A extend for 36 days, whereas BoNT/B effects last 14 days [45, 49, 50]. The use of labelled BoNTs and fragments has been instrumental not only in determining the subcellular location of LC but also in tracking their life journey, shedding light on protease lifetime (Table 1). Despite shared substrates and receptors at presynaptic terminals, the duration of synaptic block and paralytic effects of BoNT/A significantly exceeds BoNT/E [46, 49]. Research into the mechanisms underlying these differences showed that BoNT/E LC associates more readily with TRAF2, a RING finger protein implicated in ubiquitylation and proteasomal degradation [51]. To determine if BoNT/A LC could be targeted for rapid degradation to reduce its potency and action duration, chimeric SNAP25-based ubiquitin ligases have been tested, showing a significant reduction of the action duration [51]. Ubiquitin ligase domains of LC/A and LC/E were fused to red or yellow fluorescence proteins (RFP or YFP, respectively) with subcellular locations analyzed in co-transfected N18 neuroblastoma cells [51]. Most of the LC/A are localized to the plasma membrane; some are in intracellular membranes and vesicles. Surprisingly, in this study, LC/E showed a similar distribution, suggesting that the LC action longevity differences cannot be entirely attributed to subcellular localization [51]. A more recent analysis showed that treatment of submandibular gland (SMG) cells with BoNT/A increased the number of autophagosomes not colocalized with lysosome, suggesting that LC/A can change autophagic flux, disturbing autophagosome-lysosome fusion and impeding protease degradation [52].

To find out if mutations affecting the action longevity of toxins alter their turnover and breakdown, Vagin and co-workers applied site-directed mutagenesis to develop BoNT/A LC L428A/L429A fused with GFP, showing much shorter paralytic effects in mice [53]. Unlike wild-type, LC/A-GFP clustering on plasma-membrane and colocalizing with septins-2 and septin-7, LC-GFP L428A/L429A showed lower membrane clustering and reduced colocalization with septin-7. Disruption of septin oligomerization or silencing of septin-2 prevented LC/A-GFP clustering on the surface membrane and increased its degradation. The authors concluded that dileucine-mediated LC/A–septin clustering plays a crucial role in the prolonged action of LC/A and presumably in neuroparalytic activity [53]. Overall, it emerges that slower turnover of BoNT/A LC proteins contributes to the longevity of effects, with its clustering in the plasma membrane preventing rapid turnover with degradation. Nevertheless, given the conflicting results on the sub-cellular distribution of LC/A and LC/E [51], additional mechanisms might be at play, including BoNT/A disturbing autophagosome-lysosome fusion and slowing down protease degradation [52].

Wang and co-workers used his6-tagged BoNT/A-Hc linked to Atto647N maleimide to show that most endocytosed BoNT/A-Hc in hippocampal cultures was incorporated in LC3-positive autophagosomes generated in the nerve terminals, which then underwent retrograde transport to the cell soma, where they fused with lysosomes [54]. Super-resolution analysis allowed authors to visualize and characterize the dynamics of single molecules of internalized BoNT/A-Hc-Atto647N in individual axons. The pharmacological block of autophagosome formation or acidification with wortmannin or bafilomycin A_1_ inhibited the activity-dependent trafficking of BoNT/A-Hc and its breakdown [54]. Authors suggest that both the presynaptic formation of autophagosomes and the initiation of their retrograde trafficking are tightly regulated by synaptic activity.

## Internalization and Sorting of Toxins at Presynaptic Terminals

**Fig. 3:**
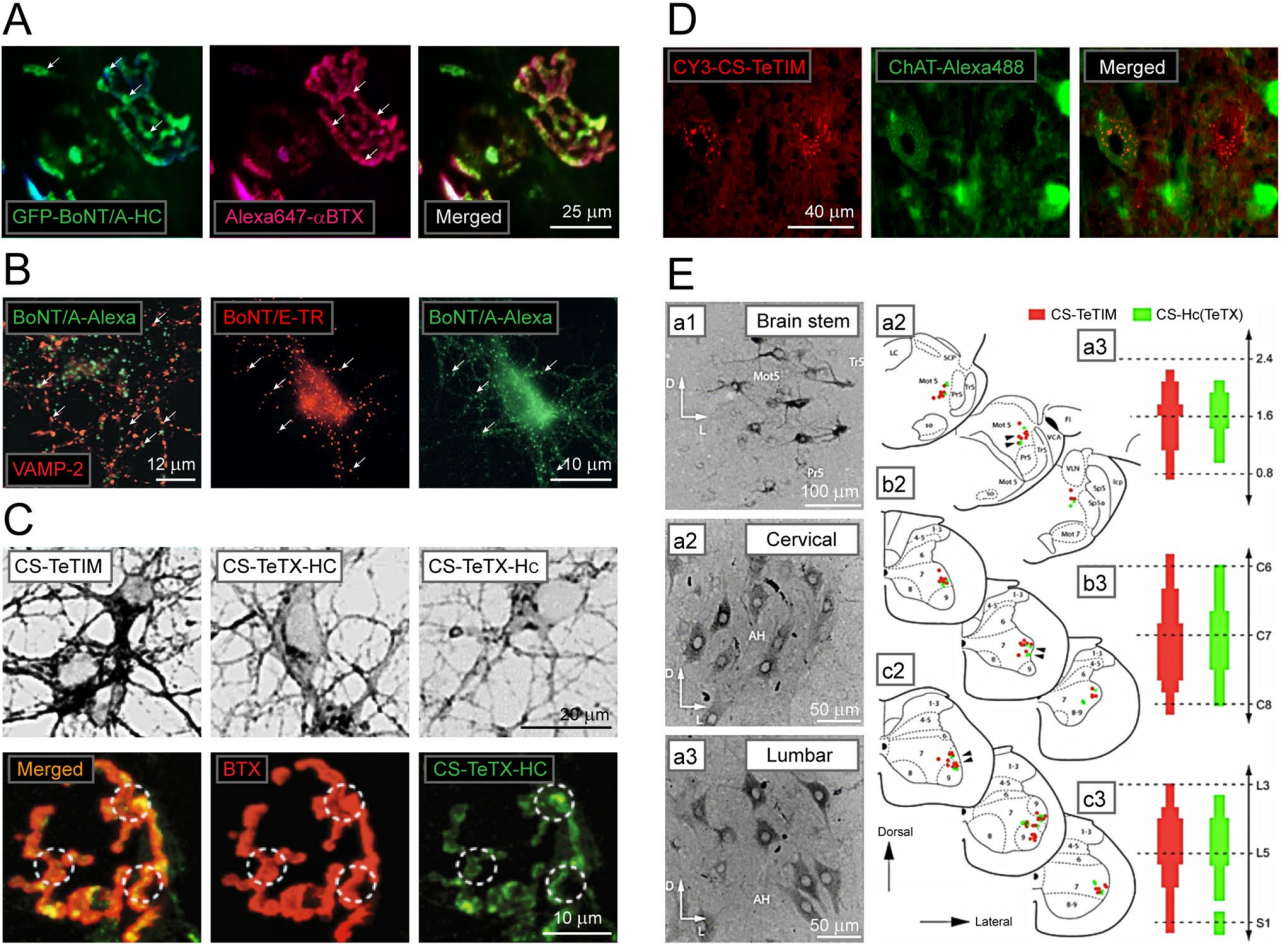
Imaging endocytosis and molecular trafficking in neurons in vitro and in vivo using BoNT and TeTX-derived optical probes. (**A**) Visualizing motor nerve endings of mouse *triangularis sterni* by GFP-BoNT/A-HC (left) and Alexa647-α-BTX targeting acetylcholine receptors of NMJ (middle). Arrows indicate areas of intense labelling at the NMJ. Merged image on the right. Adapted with permission from [57]. (**B**) Imaging synaptic contacts in neuronal cultures by BoNT/A-Alexa-488 and presynaptic marker VAMP-2 (left). Cultured neurons were exposed to 40 nM BoNT/E-TR and BoNT/A-Alexa-488 (middle and right) reveal fluorescent puncta corresponding to presynaptic terminals (arrows). Adapted permission from [63]. (**C**) Top: imaging of the uptake and transport of TeTX-derived probes CS-TeTIM, CS-TeTX-HC and CS-TeTX-HC linked with biotin-4-fluorescein in spinal cord cultures. Bottom: fluorescence confocal images of CS-TeTX-HC in the NMJ of the rat tongue 8 h post-after injection. Dashed circles – random ROI in NMJ counter-stained by rhodamine α-BTX. Adapted with permission from [33]. (**D**) Retro-axonal labelling of motor neurons by Cy3-CS-TeTIM injected in *m. gastrocnemius* (left) stained also for choline acetyltransferase (ChAT-Alexa-488) and merged image (middle and right) (in preparation). (**E**) Mapping CS-TeTIM and CS-HC(TeTx) labelled neurons in the brainstem and spinal cord. (a1-c1) Micrographs of brain stem, cervical and lumbar spinal cord showing HRP labelled CS-positive neurons (left) with (a3-c3) topographic maps of their distribution. Abbreviations: a1, a2, Mot5—motor trigeminal nucleus, Tr5 – trigeminal nerve, SCP – superios cerebellar peduncle, so – solitary tract; LS – locus coeruleus, VCA – ventral cochlear nucleus; FL-flocculus, VLN – ventrolateral nucleus, Sp5 – spinal trigeminal tract, SP5o – spinal trigeminal tract oral, Mot7 – motor nucleus, Pr5 – principal sensory trigeminal nucleus. Thick, medium and thin coloured bars indicate numbers of CS-labelled neurons. For illustration purposes, measurements at individual points are merged into continuous bars. Adapted with permission from [33]

Goodnough and colleagues made the earliest attempt to use fluor-labelled HC of BoNT/A as a nano-carrier for cargo delivery to cultured mouse neurons [55] (Table 1). After 24 h of exposure to dextran and HC conjugated with the fluorescent dyes Oregon green 488 and Cy3, punctate labelling of soma and neurites of cortical neurons was observed, with colocalization of fluorophores suggesting shared internalization and transport routes. Inhibition of the neuronal labelling by increasing the dose of BoNT/A holotoxin indicates the uptake of targeted load depends on intact SNAP-25 protein [55]. To elucidate further the mechanisms of BoNT/A HC uptake and transport of linked molecular cargo, Harper and co-workers analyzed Alexa Fluor-488 labelled BoNT/A HC using fluorescence and electron microscopy [56]. Based on the fluorophore tracking, it was found that the uptake of BoNT/A HC is activity-dependent and involves clathrin-mediated endocytosis, which is followed by sorting of the HC to late-endosomes and multivesicular bodies. Inhibition of vesicle forming GTPase dynamin activity with Dyngo-4a™ abolished Alexa Fluor-488 HC uptake. Authors suggest that dynamin inhibition at the NMJ may provide a therapeutic avenue for the treatment of botulism and other conditions caused by pathogens sharing dynamin-dependent internalization route [56]. Analysis of the effects of various sequences in the linkers between the GFP reporter and HC on  membrane binding and uptake showed that the entire BoNT/A-HC (residues 544–1295) with up to a 40 amino acid linker remained functionally active and readily taken up by cultured neurons or mouse motor nerve endings [57]. In *extensor digitorum longus* (EDL) nerve-muscle explant perfused with GFP-BoNT/A-HC (544–1295) and GFP-BoNT/A-BD (875–1295), both probes localized to the motor nerve terminals of the NMJ, similar to Alexa-647-BoNT/A full length protein [57] (Fig. 3A).

Using immunoelectron and fluorescence imaging with gold-labelled GFP antibody, the binding of eGFP-HC of BoNT/A to its receptors and internalization has been analyzed [58]. The injection of the probe into a mouse skeletal muscle caused specific labelling of the presynaptic membrane at the active zone of the NMJ, with one to two gold particles present inside small clear synaptic vesicles, in agreement with SV2 as the receptor for the uptake of BoNT/A [58–61]. This observation aligns with the reported same number of SV2 proteins per individual small synaptic vesicle [62]. Accordingly, the translocation of the LC occurs in a small synaptic vesicle and is operated by no more than two HN domains of BoNT/A. Utilizing proton-sensing pH-rodo-BoNT/A-HC, Harper and co-workers show that the endocytic pool of synaptic vesicles undergoes exocytosis and fusion with the surface membrane [56]. Notably, pH-rodo-BoNT/A-HC-loaded synaptic vesicles displayed a reduced ability for fusion with the plasma membrane compared to those loaded with pH-rodo-dextran. This observation is in line with reduced quenching of pH-rodo-BoNT/A-HC loaded nerve terminals in response to stimulation, inferring that synaptic vesicles loaded with BoNT/A are slow in entering into the next rounds of exocytosis [56]. Moreover, BoNT/A is internalized within a subset of vesicles that partially co-localize with cholera toxin B-subunit and have markedly lower VAMP2 immunoreactivity, implying significant molecular and functional heterogeneity of synaptic vesicles in respect of VAMP2 protein [56].

The difference in SNARE proteins of presynaptic terminals also contributes to their sensitivity to the toxicity of BoNTs [63]. Using fluor-labelled BoNT/A (Alexa-488) and BoNT/E (Texas-Red, TR), presynaptic terminals of hippocampal neurons have been visualized to relate synaptic vesicle dynamics and inhibitory effects of BoNT/A (Fig. 3B). Comparison of the toxic effects of BoNT/A in excitatory and inhibitory neurons has shown that the toxin more effectively inhibits the synaptic vesicle cycle and transmitter release at glutamatergic vs GABAergic neurons. The differences in toxin sensitivity do not result from unequal uptake due to SV2 discrepancies, as BoNT/A-Alexa-488 uptake upon high-K^+^ stimulation was detected equally at both types of synapses. Analysis of fluorophore-labelled LC translocation in hippocampal neurons demonstrated that LCs of BoNT/A and BoNT/E translocate equally via a bafilomycin-sensitive route from the presynaptic acidic compartments, ruling out the role of translocation to differential sensitivity of synapses to toxins. It is rather due to lower levels of SNAP-25 in inhibitory terminals, with overexpression of this SNARE protein in GABAergic neurons enhancing their sensitivity to BoNT/A, suggesting the amount of SNAP-25 as a critical factor for BoNT/A potency and toxicity at glutamatergic and GABAergic synaptic terminals [63].

## Tracking Intracellular Life Journey of Neurotoxins

In botulism, BoNTs act predominantly at their entry site at the NMJ, with some evidence suggesting trace amounts propagating retrogradely to the CNS [19, 50, 64–66]. Confocal and single-molecule live imaging of rat hippocampal neurons cultured in microfluidic devices show that the uptake of the BoNT/A-HC-Atto647N maleimide is followed by a gradual increase in the number of retrograde Atto647N carriers in axons [54] (Table 1). Using super-resolution imaging, Wang and co-authors analyzed the transport of single molecules of BoNT/A-Hc-Atto647N along individual axons. Retrograde carriers were rare in the first hour of analysis, with an apparent increase in their flux observed from 2 to 4 h after uptake. This effect was especially evident in cells that had been pulse labelled using a depolarizing high K^+^ buffer, with a ∼threefold rise in the number of BoNT/A-HC-positive endosomes compared with non-stimulated controls [54]. The number of stationary or anterograde fluorescent carriers was unchanged, with stimulation also causing no change in their motility. Over time, most of the trafficked BoNT/A-HC merged in LC3-positive autophagosomes, which gradually fused with lysosomes. The activity-dependent trafficking of BoNT/A-Hc and fusion with autophagosomes was inhibited by wortmannin or bafilomycin A_1_ [54].

Unlike botulism, tetanus is caused by the central effects of TeTX and depends on long-range retrograde and trans-synaptic trafficking. The superb capacity of TeTX to travel to the CNS has been particularly interesting for studies of molecular trafficking in MN [10, 19, 33, 67]. We have developed and analyzed the targeting and trafficking capabilities of TeTX-HC and TeTX-H_C_ fusion proteins with an adaptor core streptavidin (CS) with those of detoxified full-length TeTX –TeTIM (Fig. 3 C-E) [33]. The CS renders these recombinant molecules readily linkable with any biotinylated fluor or reporter protein. CS-TeTIM was found to be superior in binding and internalization assays in vitro and in vivo (Fig. 3C). Mapping the internalization at NMJ and retro-axonal labeling of spinal cord neurons by CS-TeTIM and CS-HC(TeTX) showed the superiority of the former, as evident from more rapid clearance from presynaptic motor nerve terminals and stronger labeling of spinal cord neurons in vivo (Fig. 3D, E). Lalli and colleagues used fluor-labelled TeTX binding fragment to elucidate cytoskeletal and motor requirements for retrograde transport [68]. TeTX HC fusion protein with glutathione S-transferase with FLASH and Alexa-488, 546, and 594 labeling showed the dependence of the process on intact microtubules and actin microfilaments [68]. The trafficking of fluor-labelled HC relies on dynein and kinesin, with dynein acting as the main molecular motor. Immunofluorescence screening with isoform-specific myosin antibodies shows colocalization of retrograde carriers with myosin Va, with axonal transport of TeTX HC significantly compromised in myosin Va knock-out mice. Live imaging of TeTX-HC-Alexa-488 revealed two groups of transport organelles—round vesicles and fast tubular structures, which do not acidify and lack markers of the classical endocytic pathway but are enriched with low-affinity neurotrophin receptor p75NTR [68]. Another study from the same group used TeTX HC fusion protein with a pH-sensitive GFP (ratiometric pHluorin) to elucidate the role of pH in regulating the sorting and trafficking of TeTX in neurons [67]. It was found that retrograde carriers display a narrow range of neutral pH values, which is kept constant during transport. In contrast, stationary pHluorin-GFP-HC loaded organelles exhibit varying pH values, ranging from acidic to neutral. This difference in acidification has been attributed to the degree of enrichment of endosomes with vacuolar (H +) ATPase, which is not present in TeTX-carrying endosomes [67]. Interestingly, although the inhibition of the vacuolar (H +) ATPase does not affect the retrograde transport of TeTX HC, it slows down the association of pHluorin-GFP-HC with long-range transport endosomes. These data indicate that the vacuolar (H +) ATPase plays an essential role in the early sorting of TeTX HC to retro-axonal carriers but does not influence subsequent trafficking along the transport route [67]. Of note, the neutral pH in TeTX-loaded endosomes also facilitates toxin escape from acidification and prevents its targeting and breakdown in degradative organelles.

Roux and co-workers analyzed the uptake and transport of TeTX-HC-GFP in MNs after its injection in a mouse *levator auris longus* (LAL) muscle [69]. A single injection of the fusion protein near the LAL muscle followed by removal and examination of the muscle showed that TeTX-HC-GFP clustered in NMJs labelled by TRITC-conjugated α-bungarotoxin (TRITC-α-BTX). Biochemical assays confirm HC-GFP binding lipid microdomains of presynaptic terminals of MNs, facilitating the uptake of the fusion protein. After 30 min, HC-GFP puncta appeared diffuse, which persisted ~ 2 h over the surface of the NMJ. Injections of GFP alone did not label the NMJ [69]. Significantly, the transport compartments of TeTX HC-GFP differed from those occupied by the cholera toxin B-subunit, suggesting potentially different routes of uptake and trafficking from those reported in cultured neurons. A recent study using live imaging of NMJ compared the targeting and transport efficacy of full-length HC of TeTX (865: TeTX-HC) and HC fragments (1066:TeTX-HC; 1093: TeTX-HC and 1109–1315, and 1109: TeTX-HC) fused with Emerald Fluorescent Protein (emGFP) [70]. All probes yielded high contrast staining of MN terminals in rodent or human muscle explants without functional changes, as shown by electrophysiological recordings of endplate potentials from mouse muscles. Fibre-optic live confocal endomicroscopy confirmed emGFP-TeTX-HC-Alexa647-α-BTX double labeled terminals, which were absent from denervated as well as in degenerating NMJs of SOD1G93A amyotrophic lateral sclerosis (ALS) mouse muscle [70].

## Visualizing Neural Circuits and Connections

**Fig. 4:**
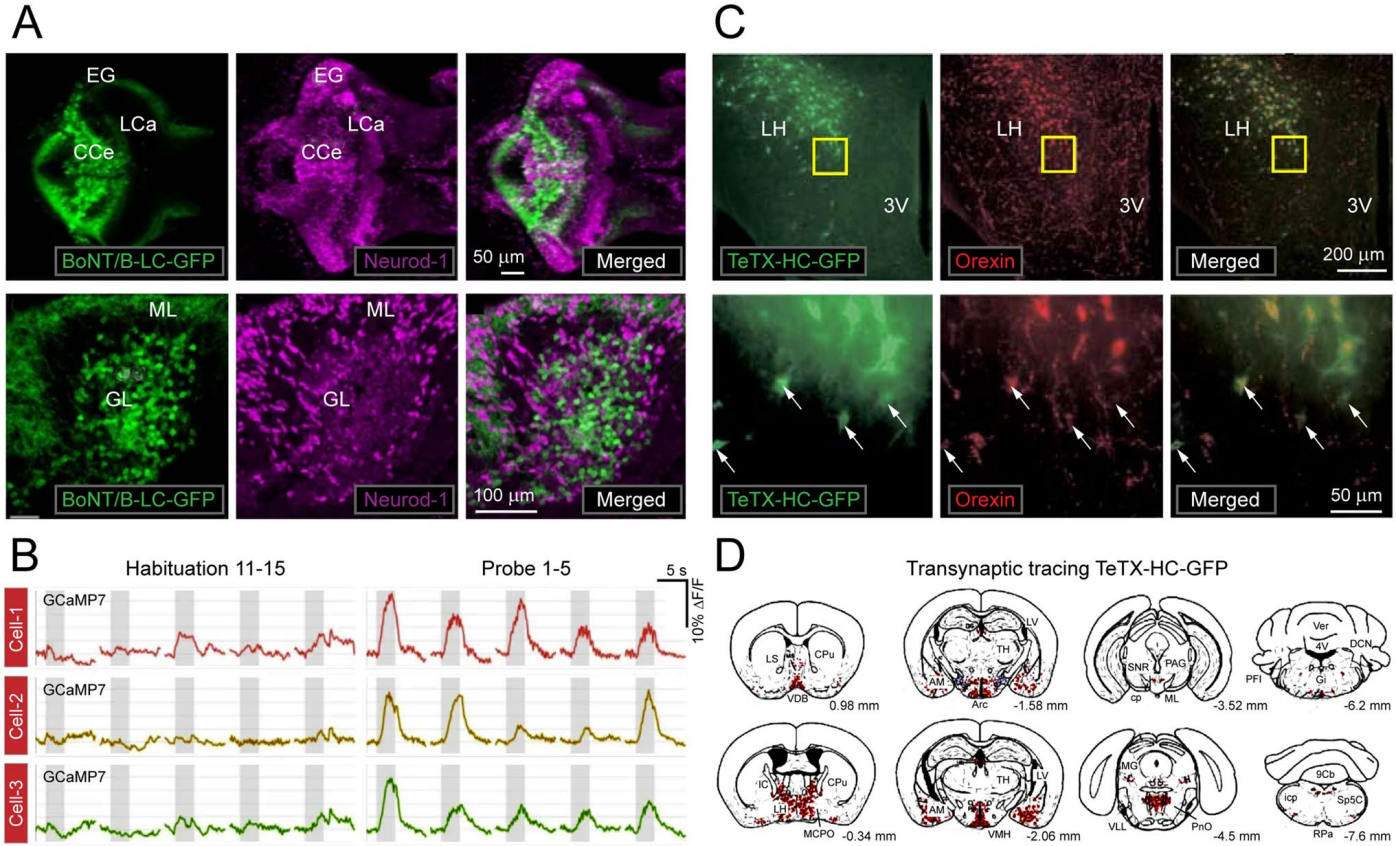
Imaging selected neuronal groups and connectivity in the CNS using toxin-derived optical probes. (**A**) BoNT/B-LC-GFP expression in 20-days post-fertilization (dpf) granule cell-silenced larvae of zebra fish. Immunostaining of the brain (dorsal view, top row) and sagittal sections (bottom row) with anti-GFP (green) and Neurod1 (magenta, nuclear marker) antibodies. About half of the mature granule cells express BoNT/B-LC-GFP. EG – eminenta granularis; LCe – lobus caudalis cerebelli; CCe – corpus cerebelli; GL, granule cell layer; ML, molecular layer. (**B**) Sample recordings of the neuronal activity using GCaMP7a Ca^2+^ sensor with measurements before and during-after stimulation in a single larva during the habituation (11th–15th trials) and probe (1st–5th trials) sessions. The graphs depicts the ΔF/F. Gray boxes indicate the timing of the CS presentation. Adapted with permission from [71]. (**C**) Transgenic mice expressing TeTX-HC-GFP in orexin neurons showing double-label immunofluorescence for GFP and Alexa594 conjugated with anti-GFP IgG in the lateral hypothalamus (LH). (**D**) Mapping TeTX-HC-GFP positive neurons from rostral to caudal planes of the brain coronal sections revealed by immunohistochemical staining. Abbreviations: VDB—vertical limb of the diagonal band; LS—lateral septum; CPu—caudate putamen; IC—internal capsule; LH—lateral hypothalamus; MCPO—magnocellular preoptic nucleus; LV- lateral ventricle; TH—thalamus; AM—amygdala; Arc—arcuate hypothalamic nucleus; VMH, ventromedial hypothalamus; SNR—substantia nigra; ML—medial lemnisci; PAG—periaqueductal gray; cp—cerebral peduncle; VLL—ventral nucleus of lateral lemniscus; PNO—pontine reticular nucleus, oral part; VER—vermis; 4 V—forth ventricle; DCN- deep cerebellar nucleus; PFI—paraflocculus; Gi—giganto-cellular reticular nucleus; 9Cb—nine cerebellar lobules; icp—inferior cerebellar peduncle; Sp5C—spinal trigeminal nucleus caudal; RPa—raphe pedunculus nucleus. Adapted with permission from [73]

Utilizing cell-type specific expression of GFP fused with LC of BoNT/B (BoT/B-LC-GFP), the effects of presynaptic silencing of neurons on the functionality of cerebellar circuits has been analyzed in fear conditioning model of zebrafish [71] (Fig. 4A) (Table 1). The granule-cell-specific Gal4 line gSA2AzGFF152B25 was crossed with the Tg(UAS:BoT/B-LC-GFP) yielding GFP and BoNT/B LC (BoT/B-LC-GFP) expression exclusively in cerebellar granule cells, inhibiting Gal4-dependent synaptic release of glutamate. The substantial decrease of Ca^2+^ transients of GCaMP7a in larvae cerebellum agrees with the global silencing of activity by BoT/B-LC-GFP expressed in granule cells [71] (Fig. 4B). Behavioural studies at 20 days post-fertilization (dpf) showed that selective silencing of granule cell activity in the corpus cerebelli did not suppress the fear-conditioned bradycardia but prolonged the duration of the response. The approach allowed imaging of cerebellar neurons and the effects of silencing to the conditioned stimulus, with their activity enhanced during fear conditioning and reduced by the repetition of the unpaired stimulus [71].

Kissa and co-workers used grafting of GFP-TeTX HC transfected neurons to monitor the transport of GFP fluorescence [72]. It was shown that following TeTX HC synthesis in grafted neurons, the fusion protein can be released and internalized by neighbours around the injection site. Utilizing the adeno-associated viral (AAV) vector to express the fusion protein in neurons, it was found that transduced neurons can deliver the fusion protein specifically into connected partners, demonstrating that synaptic transfer in the CNS can be visualized with GFP-TeTX-HC. Applying a similar approach to the expression of GFP TeTX-HC in orexin neurons of the hypothalamus, Sakurai and co-workers visualized orexin profiles of the lateral hypothalamus and retrogradely labelled upstream neurons with synaptic connections [73] (Fig. 4C, D). GFP-positive neurons were observed in multiple brain regions, including the basal forebrain, ventrolateral preoptic nucleus, median raphe and paramedian raphe nuclei, which play a crucial role in regulating the sleep and wakefulness states [73] (Fig. 4D). The method allowed screening of the response of orexin neurons to cholinergic agonist carbachol, which excited nearly one-third of orexin neurons and inhibited the rest. These data uncover complex neurochemical mechanisms regulating the activity of orexin neurons and provide insights into pathways that control sleep-wakefulness, vigilance states, arousal, and appetite [73]. A similar approach using TeTX-HC-GFP assisted mapping synaptic connections in developing and mature mammalian CNS [74]. Based on the transsynaptic transfer of TeTX-HC-GFP, it was possible to visualize cells interconnected via monosynaptic contacts. Using the targeted expression of GFP and lacZ reporter, the authors were able to discriminate neurons that produce the tracer from those that have acquired it via synaptic uptake to reconstruct connections and dynamics of neural circuits during the embryonic and post-natal development [74]. When expressed under calbindin promoter, TeTX-HC-GFP enabled visualization of well-defined neural circuits and projections in the cerebellum, including synaptic inputs of granule and basket cells to Purkinje neurons. In the same vein, a robust and reproducible expression of GFP was achieved in the olfactory bulb, cerebral cortex, cerebellum, hippocampus, hypothalamus, and striatum of multiple preclinical models [74].

In another neurodevelopmental study, expression of TeTX LC fused with CFP was used to determine how chronic presynaptic silencing affects the formation of synaptic connections and the integration of neurons into developing networks. Hippocampal neurons expressing TeTX LC-CFP were co-cultured with naïve neurons [75]. Despite the complete block of evoked glutamatergic transmission, silenced neurons formed as many presynaptic terminals as their active neighbors when grown together on glial micro-islands. Of note, silent glutamatergic inputs failed to recruit the GluR1 subunit of AMPA-type receptors as efficiently when competing with naïve neighbours. As a result, the ratio of GluR1 within synaptic puncta was reduced compared to active intact synaptic terminals [75]. The synaptic level of the AMPA receptor subunits GluR2 or GluR2/3 and the PSD95 family scaffolding proteins in CFP-expressing neurons remained unchanged, implying that during development, activity-dependent release of glutamate regulates the composition and stoichiometry of AMPA receptor subunits on a synapse-by-synapse basis [75].

## Summary and Future Directions

Over millennia, the lethal effects of biological toxins prompted not only reverence but also curiosity and aspiration to use them as weaponry and medicine. In addition to harnessing pharmacological and therapeutic actions, advances in biomedical research and recombinant technologies enabled their application for targeting selected neuron types and receptors, interfering with specific functional and molecular processes. Combined with the expanding toolkit of fluorophores and reporter proteins, targeting and delivery capabilities of biological toxins have empowered a wide range of new applications in molecular neuroimaging.

Since the discovery of BoNT binding to motor nerve terminals of the NMJ [31], BoNTs and TeTX have become instrumental in unravelling the mechanisms of transmitter release at peripheral and central synapses. Combined with fluorescent nanocomposites and reporter proteins, BoNTs, TeTX and their fragments have also grown into an integral part of the neuroimaging toolkit, prompting discoveries in molecular, cellular, and developmental neurobiology. Capitalizing on superb neurotropism, specificity, and distinct intracellular transport routes of BoNTs and TeTX, optical probes derived from these toxins also shed light on mechanisms and pathways of their uptake at presynaptic terminals, action site, potency and longevity effects. Along with the labelling of synaptic terminals, the block of transmission by toxins also helped elucidate the role of neural activity in developing synaptic connections in different functional and behavioral contexts. Finally, toxin-derived probes have elucidated the intricate trafficking routes and mechanisms of BoNTs and TeTX and their fragments, empowering multiple applications in experimental research (Table 1).

The growing use of BoNTs and TeTX-derived optical probes for neuroimaging has also revealed significant challenges. The risks of residual toxicity of LC proteases are of primary concern for preclinical studies in animal models and their potential clinical use. Loss of targeting and delivery capabilities and associated off-target effects caused by added labels and reporter proteins is another major obstacle to their applications. Additional challenges are set by the immunogenicity and bioavailability of toxin-derived probes owing to their biological activity and large size. Finally, integrated fluor labels and reporter proteins can cause phototoxicity by releasing harmful by-products. It is important to note that in addition to the restrictions of biological nature, the use of toxin-derived optical probes can also be constrained by the physical properties of the light with its limited penetration into biological specimens, confining the high-resolution interrogation to the surface of the examined specimen. Addressing these challenges with further optimization of probes should facilitate harnessing their full potential in the foreseeable future.

## Data Availability

Not applicable**.**

## Abbreviations

BoNTs: Botulinum neurotoxins
TeTX: Tetanus toxin
SNARE: Soluble N-ethylmaleimide-sensitive factor activating protein receptor
HC: Heavy chain
HN: N-terminal translocation domain of HC
LC: Light chain
NMJ: Neuromuscular junction
MN: Motor neurons
CNS: Central nervous system
GFP: Green fluorescence protein
AA: Amino acid
YFP: Yellow fluorescence protein
eGFP: Enhanced GFP
DAS: Digit Abduction Scoring
RFP: Red fluorescence protein
SMG: Submandibular gland
SV2: Synaptic vesicle protein 2
VAMP2: Vesicle associate membrane protein 2
GABA: γ-Aminobutyric acid
ATP: Adenosine 3-phosphate
CFP: Cyan fluorescence protein
AMPA: α-Amino-3-hydroxy-5-methyl-4-isoxazolepropionic
PSD95: Post-synaptic density 95 protein
